# Pneumococcal Polysaccharide Vaccination Elicits IgG Anti-A/B Blood Group Antibodies in Healthy Individuals and Patients with Type I Diabetes Mellitus

**DOI:** 10.3389/fimmu.2016.00493

**Published:** 2016-11-14

**Authors:** Wendelin Wolfram, Kai M. T. Sauerwein, Christoph J. Binder, Nicole Eibl-Musil, Hermann M. Wolf, Michael B. Fischer

**Affiliations:** ^1^Clinic for Blood Group Serology and Transfusion Medicine, Medical University of Vienna, Vienna, Austria; ^2^Immunology Outpatient Clinic, Vienna, Austria; ^3^Department of Laboratory Medicine, Medical University of Vienna, Vienna, Austria; ^4^Rudolfstiftung Hospital of the City of Vienna, Vienna, Austria; ^5^Sigmund Freud Private University – Medical School, Vienna, Austria; ^6^Department for Health Science and Biomedicine, Danube University Krems, Krems, Austria

**Keywords:** pneumococcal polysaccharide vaccine, 23-valent pneumococcal polysaccharide vaccine, anti-blood group A/B antibodies, surface plasmon resonance, isoagglutinins, natural antibodies

## Abstract

**Hypothesis:**

Blood group antibodies are natural antibodies that develop early in life in response to cross-reactive environmental antigens in the absence of antigen encounter. Even later in life structural similarities in saccharide composition between environmental antigens such as bacterial polysaccharides and blood group A/B antigens could lead to changes in serum levels, IgM/IgG isotype, and affinity maturation of blood group anti-A/B antibodies. We addressed the question whether immunization with pneumococcal polysaccharide (PnP) vaccine Pneumo 23 Vaccine “Pasteur Merieux” (Pn23) could have such an effect in patients with type I diabetes mellitus (DM I), an autoimmune disease where an aberrant immune response to microbial antigens likely plays a role.

**Methods:**

Anti-PnP IgM and IgG responses were determined by ELISA, and the DiaMed-ID Micro Typing System was used to screen anti-A/B antibody titer before and after Pn23 immunization in 28 healthy individuals and 16 patients with DM I. In addition, surface plasmon resonance (SPR) technology using the Biacore^®^ device and a synthetic blood group A/B trisaccharide as the antigen was applied to investigate IgM and IgG anti-A/B antibodies and to measure antibody binding dynamics.

**Results:**

All healthy individuals and DM I patients responded with anti-PnP IgM and IgG antibody production 4–6 weeks after Pn23 immunization, while no increase in blood group anti-A/B antibody titer was observed when measured by the DiaMed-ID Micro Typing System. Interestingly, isotype-specific testing by SPR technology revealed an increase in blood group anti-A/B IgG, but not IgM, following Pn23 immunization in both patients and controls. No change in binding characteristics of blood group anti-A/B antibodies could be detected following Pn23 vaccination, supporting the assumption of an increase in IgG antibody titer with no or very little affinity maturation.

**Conclusion:**

The study provides evidence for epitope sharing between pneumococcal polysaccharides and blood group ABO antigens, which leads to a booster of blood group anti-A/B antibodies of the IgG isotype after Pn23 immunization in healthy individuals. Manifest autoimmunity such as present in DM I patients has no additional effect on the cross-reactive antibody response against pneumococcal polysaccharides and blood group antigens.

## Introduction

Natural antibodies are produced in the absence of overt external antigenic stimulation early in life in all healthy individuals with a functional immune system, presumably as a product of germline gene segment assembly (1). Therefore, they show low affinity to many microbial pathogens and certain cross-reactivity, even to some self-antigens (1–4). Antibodies to carbohydrate epitopes share certain characteristics with natural antibodies, e.g., the absence of extensive class switching and affinity maturation due to absent T-cell help (5). Isoagglutinins, i.e., antibodies against carbohydrate epitopes that form the AB0 antigens on red blood cells (RBCs), are considered prototypic natural antibodies that were shown to be compromised in patients with immunodeficiency (1, 4, 6). Their appearance can be explained by inapparent stimulation particularly from intestinal bacteria because antigens cross-reactive with blood group polysaccharides are widely distributed in the environment (1, 7, 8). Natural anti-ABO antibodies have long been considered to be predominantly of the IgM isotype, but antibodies of the IgG isotype are also produced, especially later in life, e.g., after alloimmunization in pregnancy or ABO-incompatible blood transfusions (1). In the case of ABO blood groups, individuals with blood group A develop an immune response to B-like antigens of microorganisms and environmental antigens like plants containing these epitopes (1, 9). B blood group individuals develop anti-A antibodies, O blood group individuals produce both anti-A and anti-B antibodies, while AB blood group subjects have neither anti-A nor anti-B because they express both antigens on their RBCs.

The human ABO blood group antigens are oligosaccharide moieties formed on precursor backbones by glycosyltransferases (4, 9, 10). Blood group O individuals have carbohydrate structures named H-antigen terminated in the sequence alpha-Fuc(1,2)Gal. The blood group A antigen is then formed from the H-antigen by an *N*-acetylgalactosaminyltransferase that uses a UDP-*N*-acetylgalactosamine (GalNAc) donor, and the blood group B antigen is formed by a galactosyltransferase that uses a UDP-galactose (Gal) donor to convert the H-antigen (4). The human A and B blood group antigens differ from each other only in the substitution of an acetamino for a hydroxyl group on the terminal saccharide residue, and this substitution can be differentially recognized by specific antibodies (1, 4, 11, 12). Interindividual variations in the titer of anti-blood group ABO antibodies exist, representing modulation of the production of these natural antibodies in response to cross-reactive environmental antigens, e.g., through immunization (1). B cells could thus recognize shared carbohydrate epitopes due to structural similarities in saccharide composition, e.g., between microbial polysaccharides (PnPs) and blood group A/B antigens, which could also have an effect on blood group anti-A/B specific antibody production after PnP-immunization. Previous findings indicate shared epitopes between blood group oligosaccharides and pneumococcal capsular polysaccharides, as antibodies cross-reacting with human erythrocytes and pneumococcal antigens have been described (7). Furthermore, it was shown that cross-reactive carbohydrate determinants (CCDs) contained in blood group antigens and microbial polysaccharides can be targeted by immunoglobulins of different isotypes, in particular by IgE, but also by IgG antibodies (13).

In addition to their role as a first line of defense against infection, natural antibodies have been shown to play an important role in the initiation of autoimmunity (2, 8, 14–16). Natural antibodies reacting with self-antigens were initially considered to represent breakdown of tolerance, thus functioning as a template for the generation of pathogenic autoantibodies produced through a process of clonal selection, somatic hypermutation, and class switching driven by antigen (14). More recently, these antibodies have been attributed an additional role in the prevention of autoimmune disease, e.g., by inhibiting activation of the adaptive immune system by molecules released from apoptotic cells that could facilitate autoimmune events (15, 16). Formation of natural antibodies with high cross-reactivity could be regulated differently in healthy individuals as compared to individuals with an autoimmune disease such as type 1 diabetes mellitus (DM I) known to present with abnormalities in antibody formation (17). In addition to high-risk HLA genes, environmental factors are thought to play an important role in the pathogenesis of DM I, such as pancreatic virus infection, T cell-mediated and autoantibody-mediated molecular mimicry, and bystander activation of autoreactive lymphocytes by proinflammatory cytokines derived from dendritic cells activated by infection-related stimuli through innate pattern recognition receptors such as TLR (18–21). A single causative infectious agent for DM I autoimmunity has not been found, pointing toward the possibility that different infectious agents are capable of non-specifically enhancing the likelihood of autoimmunity. In addition to virus infection (19, 20), bacterial infection might play a role in the development of DM I, as antibodies against mycobacterial and proinsulin epitopes (22) as well as beta cell antigens (23) show cross-reactivity in children with new onset DM I. While human IDDM autoantigens are typically T-dependent protein antigens, microbial polysaccharides have been shown to modulate the autoimmune response leading to DM1 (24). Innate signaling *via* TLR and MyD88, pathways critical for T cell-independent (TI) immune defense (25), can reverse anergy in autoreactive B cells, suggesting that environmental factors associated with bacterial infection and inflammation may alter tolerance (26). Concerns that vaccination might lead to the development of autoimmune disease such as DM I have been raised (27), which could also relate to polysaccharide vaccines, as molecular mimicry in bacterial polysaccharide components is capable of inducing autoreactive antibodies, e.g., against some blood group antigens or neuronal gangliosides (28). In the present study, we investigated whether vaccination with Pneumo 23 Vaccine “Pasteur Merieux” (Pn23) has an effect on blood group ABO antibodies in healthy individuals and in patients with DM I. To screen for blood group anti-A/B antibodies, we used the commercially available DiaMed-ID Micro Typing System based on erythrocyte agglutination (6). To measure blood group-specific anti-A/B antibodies in healthy individuals and in patients with DM I in an isotype-specific manner, we used surface plasmon resonance (SPR) technology and synthetic A/B trisaccharides bound *via* amine-coupling to the CM5 chip (6, 11, 12). SPR using blood group-specific A/B synthetic trisaccharides is the ideal technology to investigate a potential IgM to IgG isotype shift and to investigate changes in binding characteristics of the relevant blood group anti-A/B antibodies under near to physiological conditions in real time.

## Materials and Methods

### Vaccination of Healthy Individuals and Patients with Type I DM and Measurement of Anti-PnPs Antibodies

During a clinical study published previously (17), healthy individuals (*n* = 39) and patients with type I DM (*n* = 20) were immunized with the PnPs vaccine Pn23 (a 23-valent vaccine containing pneumococcal capsular polysaccharide serotypes 1, 2, 3, 4, 5, 6B, 7, 8, 9N, 9V, 10A, 11A, 12F, 14, 15B, 17F, 18C, 19A, 19F, 20, 22F, 23F, and 33F, Pasteur Merieux Connaught, Lyon, France) after informed consent was obtained (17, 29). Type I DM patients were, on average, 16 ± 7.4 years (SD) at the onset of disease, and at that time they tested positive for GAD autoantibodies. Age at initiation of the vaccination study was 31.7 ± 8.8 years (SD). For a detailed description of patients and controls, see Ref. (17). Blood samples were drawn 4–6 weeks after vaccination, the serum was immediately separated from the cellular blood component, dispensed into 500 μl aliquots, and stored until analysis in a freezer at −20°C. For this follow-up study, serum aliquots stored at −20°C were still available from 28 healthy controls and 16 DM I patients; selection of this subset of the previous study was only because no more serum was available from the other controls and DM I patients of the original study. Anti-PnPs antibodies were determined by a home-made ELISA detecting all serotypes as previously described (17, 30).

### Determination of Anti-A/B Antibody Titers by the DiaMed-ID Micro Typing System

Titers of blood group anti-A and anti-B antibodies were determined by the DiaMed-ID Micro Typing System (Bio-Rad Lab. Inc., Hercules, CA, USA) used according to the manufacturer’s protocol. The ID-Card 50520 (NaCl) was used to measure IgM, while the ID-Card 50531 [LISS/Coombs containing poly-specific anti-human gamma globulin (AHG) serum] was used to determine IgG in addition to IgM. The serum samples were serially twofold diluted with 0.9% saline solution starting with 500 μl serum and 500 μl saline solutions. Also, 25 μl of each dilution were pipetted into a single column of the microtube gel card, and 50 μl of DiaMed-ID-DiaCell ABO/I-II test cell suspensions A_1_ or B (Bio-Rad Lab.) was added. ID-Card 50520 (NaCl) cards were incubated at room temperature for 15 min, the ID-Cards (LISS/Coombs) at 37°C, and thereafter centrifuged in a DiaMed-ID-Centrifuge 24 S for 10 min at 910 rpm. Results were read immediately after centrifugation and were subdivided into positive and negative results. The titer that led to visible agglutination of erythrocytes dispersed in the gel was selected positive. In an individual with blood group O, both anti-A and anti-B antibody titers were used for statistical analysis, while in an individual with blood group A, only anti-B antibody titers, and in an individual with blood group B, only anti-A antibody titers were used for statistical analysis; none of the study individuals had blood group AB.

### Determination of Anti-A/B Antibodies by Surface Plasmon Resonance

**Figure 1:**
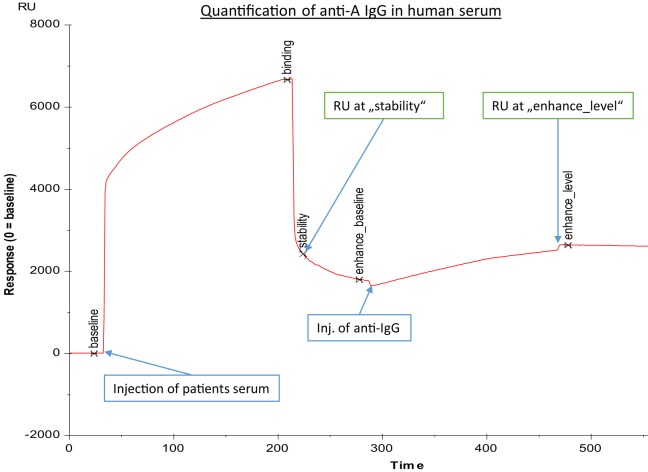
**Exemplary binding curve of quantification of anti-A IgG antibodies in human serum, as generated by BIAevaluation^®^ software (GE Healthcare)**. Injection of human serum was followed by a second injection of anti-IgG; response units (RU) at “stability” were recorded to assess the concentration of anti-A blood group antibodies, while response units at “enhance_level” were representative for anti-A IgG concentration. The *x*-axis gives time in seconds, and the *y*-axis represents response units (RU).

**Figure 2:**
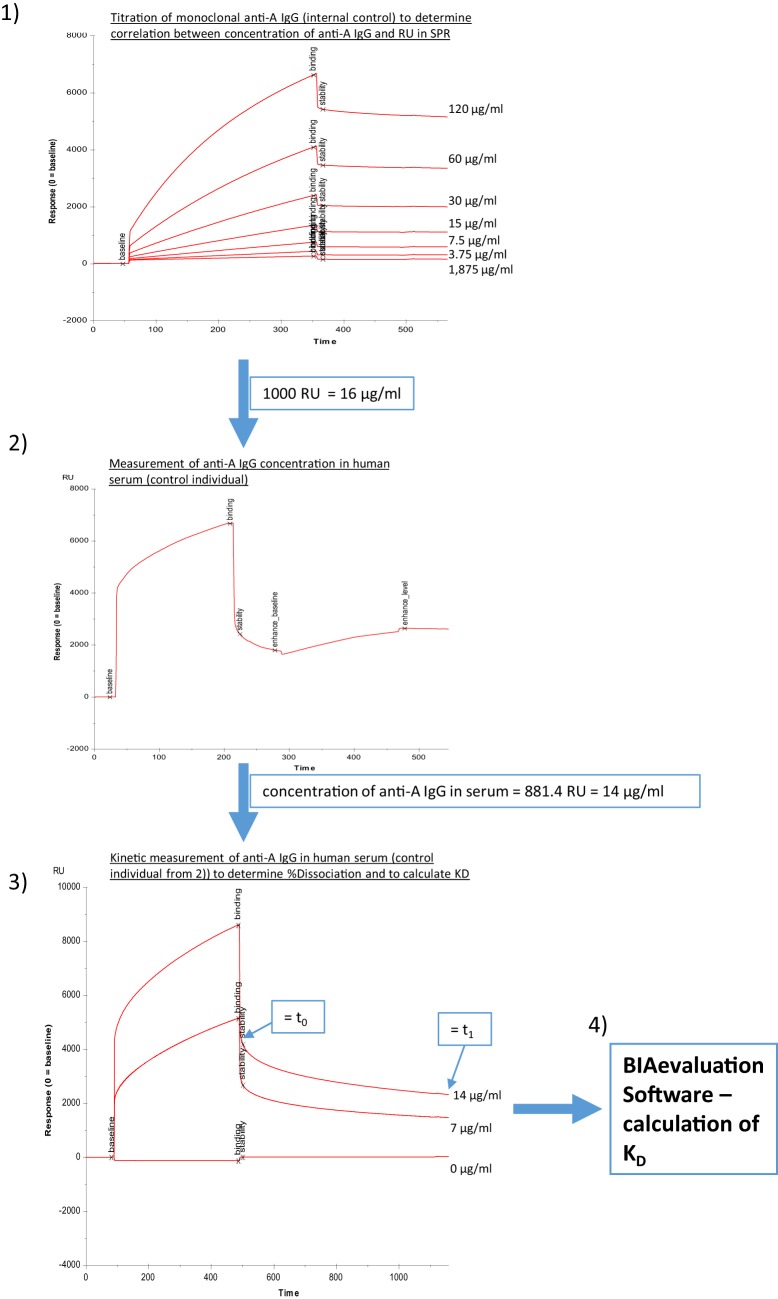
**Determination of binding characteristics of anti-A IgG antibodies in human serum**. Panel (1) different concentrations of monoclonal anti-A IgG (internal control) were injected to correlate response units to micrograms per milliliter, (2) anti-A IgG levels in human serum were determined utilizing a second injection of anti-IgG (Sigma), (3) different dilutions of human serum were measured without addition of second step reagent, and binding curves were recorded for a total of 1,200 s, (4) an estimate of *K*_D_ was calculated using the BIAevaluation Software (GE Healthcare) with input of the concentrations of anti-A IgG determined in steps (2) and (3) as described in Section “Materials and Methods.”

The erythrocyte aggregation assays based on the DiaMed-ID Micro Typing System are semiquantitative assays that cannot determine in an isotype-specific way a subtle increase of anti-A/B IgG and/or IgM antibodies against blood group A/B antigens. Therefore, we used SPR to measure anti-A/B antibodies as previously described (6, 11, 12). Amine-labeled blood group A and B trisaccharides (Dextra Laboratories Ltd., Reading, UK) were immobilized on two-channel sensor chips CM5 (General Electric Health Care, Freiburg, Germany) using standard protocols (31, 32). In brief, the CM5 sensor chip surface was activated with 100 μl 0.05 mol/l *N*-hydroxy-succinimide and 0.2 mol/l *N*-ethyl-*N*′-dimethylamino-propylcarbodiimide injected into the buffer stream of HBS-EP (0.01 mol/l HEPES buffer, pH 7.4, containing 0.15 mol/l NaCl, 3 mmol/l EDTA, and 0.005% surfactant P20) as described previously (6). The buffer stream was passed through FC-1 and FC-2 of the two-channel Biacore^®^ X instrument (GE Health Care) at a flow rate of 5 μl/min for 20 min. Subsequently, 15 μl of a blood group A/B trisaccharide amine derivative solution at a concentration of 1 mg/ml, prepared with borate (pH 8.5), was injected into the buffer stream and passed through FC-1 at a flow rate of 5 μl/min and was halted after injection of 7 μl for 2 h; FC-2 served as a blank control. Residual active ester groups on the sensor surface were then deactivated by injecting 100 μl of 1 mol/l ethanolamine–HCl (pH 8.5) into the buffer stream and passing it through FC-1 and FC-2, and by passing the buffer stream through the cell until a stable baseline was achieved. To measure anti-blood group A/B antibodies, serum was diluted by half with the HBS-EP, and 100 μl of the diluted plasma samples were passed through FC-1 and FC-2 at a flow rate of 20 μl/min for 5-min duration. When the association phase was finished, binding of antibody was recorded and is expressed as resonance units (RU at “stability” in Figure 1, corresponding to RUt0 in Figure 2, panel 3). After each measurement, the antibodies bound to the blood group trisaccharides were removed with 50 μl of 50 mmol/l NaOH buffer at a flow rate of 60 μl/min for 50 s and the chip regenerated to reach the same baseline as prior to the measurement. The amounts of anti-A/B antibody that associated with the blood group A/B trisaccharide antigen immobilized on the sensor chip surface were obtained by subtracting the FC-2 value from the FC-1 value.

Alternatively, a four channel Biacore^®^ T200 device (kindly provided by Florian Koelle, GE Health Care) was used, which enabled us to measure both anti-A and anti-B antibodies on one chip surface (CM5-chip) at the same time. The amount of trisaccharides and the coupling procedure remained similar to the two-channel system used previously, except the contact time was adapted to 600 s and the flow rate to 10 μl/min. Flow cell one (FC-1) and three (FC-3) were immobilized without trisaccharides and served as blank during binding analysis. FC-2 was immobilized with 1 mg/ml blood group A trisaccharide and FC-4 with 1 mg/ml blood group B trisaccharide. For binding analysis, serum was diluted by half with HBS-EP and injected in FC-2 and FC-4 for 180 s at a flow rate of 10 μl/min. The flow-path was 2–1 and 4–3. To determine IgM and IgG levels of A- and B-bound antibodies, anti-human IgG Abs [polyclonal aHIgG (γ-chain), Sigma-Aldrich, St. Louis, MO, USA] and anti-human IgM [polyclonal aHIgM (μ-chain), Sigma-Aldrich, St. Louis, MO, USA] were injected for 180 s at a flow rate of 10 μl/min directly after the serum sample. For measurement of anti-A/B antibodies RU at report point “stability” (see Figure 1, corresponding to RUt0 in Figure 2, panel 3) and for levels of IgM/IgG anti-A/B antibodies levels at report point “enhance_level” (see Figure 1) relative to baseline were recorded. After determination of levels of IgM and IgG anti-A/B antibodies, the chip was regenerated twice with 50 mmol NaOH for 30 s at a flow rate of 10 μl/min with a stabilization period of 5 s after the second regeneration to reach the same baseline as prior to the measurement. For kinetic measurement, an association time of 200 s and dissociation time of 800 s was chosen. Dissociation of bound anti-A/B antibodies over time as a semiquantitative correlate for the strength of antibody binding was calculated according to the following formula: % dissociation = 100 − [RU at t1 (800 s)/RU at t0 (200 s)] × 100 (see Figure 2, panel 3 as an example for measurement of RU at t0 and at t1). To compute an affinity constant *K*_D_ the concentration of IgG antibodies in microgram per milliliter must be known, thus RUs for different concentrations of monoclonal anti-A/B IgG (clone 9A, Abcam, Cambridge, MA, USA; clone NaM87-1F6, BD Pharmingen, San Jose, CA, USA) were measured (Figure 2, panel 1). This enabled us to correlate an increase in response units derived from anti-A/B IgG measurements in patient serum with microgram per milliliter of anti-A/B IgG antibody by regression analysis in order to calculate the respective concentration of IgG anti-A/B (Figure 2, panel 2). Subsequently two dilutions of patient serum – one of them diluted 1:2 – and a blank were measured (Figure 2, panel 3), and the resulting binding curves were used to calculate *K*_D_ (computed as *K*_D_ = *K*_off_/*K*_on_) with the BIAevaluation^®^ software from GE Healthcare. To fit the association/dissociation curve, a 1:1 binding model was chosen. The molecular weight of the analyte was assumed to be 150,000 Da (IgG). To assess the quality of the calculations, controls within the software were used. No external calculations were performed nor parameters added. Furthermore, as an internal control to test before each measurement was started, anti-blood group A or B IgM mAbs (anti-A murine monoclonal Abs clone MH04 and A3D3, anti-B murine monoclonal Abs clone NB1.19, NB10.5A5, and NB10.3B4, Ortho-Clinical Diagnostics, Neckargemünd, Germany), and anti-blood group A IgG mAb (clone 9A, Abcam, Cambridge, MA, USA; clone NaM87-1F6, BD Pharmingen, San Jose, CA, USA) were used to guarantee optimal chip performance and full recovery. For statistical analysis, both anti-A and anti-B antibody titers (RU) were used in an individual with blood group O, while in an individual with blood group A, only anti-B antibody titers, and in an individual with blood group B, only anti-A antibody titers were used for statistical analysis; none of the individuals in this study had blood group AB.

### Statistical Analysis

Statistically significant differences between study groups were calculated using the non-parametric two-tailed Mann–Whitney *U*-test. Results are depicted using box plot diagrams, with the median represented by a cross, the interquartile range (IQR) represented by the box, and 5- and 95-percentile values represented by the whiskers.

## Results

### Anti-PnPs Response in Healthy Individuals and in Patients with Type I DM after Pneumovax^®^23 Immunization

**Figure 3:**
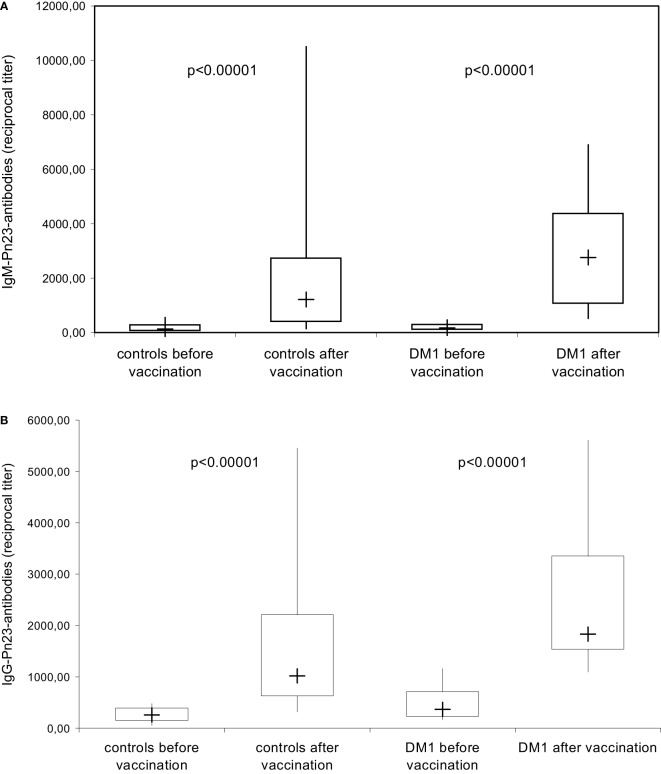
**IgM/IgG antibody response to pneumococcal polysaccharides after immunization with Pn23 vaccine**. Healthy individuals (*n* = 28) and patients with type I DM (*n* = 16) were immunized with the PnPs vaccine Pneumo 23 Vaccine “Pasteur Merieux” (Pn23), and blood samples were drawn 4–6 weeks after vaccination. Antibodies against pneumococcal polysaccharides (anti-PnPs) were determined by ELISA as described in Section “Materials and Methods” and presented as IgM–Pn23-antibody response **(A)** or IgG–Pn23-antibody response **(B)**. Results are depicted using box plot diagrams, with the median represented by a cross, the interquartile range (IQR) represented by the box, and 5- and 95-percentile values represented by the whiskers; statistically significant differences between study groups were calculated using the non-parametric two-tailed Mann–Whitney *U*-test.

The anti-PnPs antibody response prior and 4–6 weeks after immunization with the unconjugated PnPs vaccine Pn23, a 23-valent vaccine, was determined by ELISA in an isotype-specific manner in 28 healthy individuals and 16 patients with DM I. All healthy individuals included in this study showed a significant IgM (Figure 3A) and IgG (Figure 3B) anti-PnPs antibody response following vaccination. The results confirm previous findings in patients with type I DM (17) showing a normal antibody response to unconjugated pneumococcal polysaccharide, which is likely TI, while primary antibody responses to T-cell-dependent antigens are impaired (17).

### Increase in Blood Group IgG Anti-A/B Antibodies in Healthy Individuals and in Patients with Type I DM after Pneumovax^®^23 Immunization as Determined with the Biacore^®^ Device

**Figure 4:**
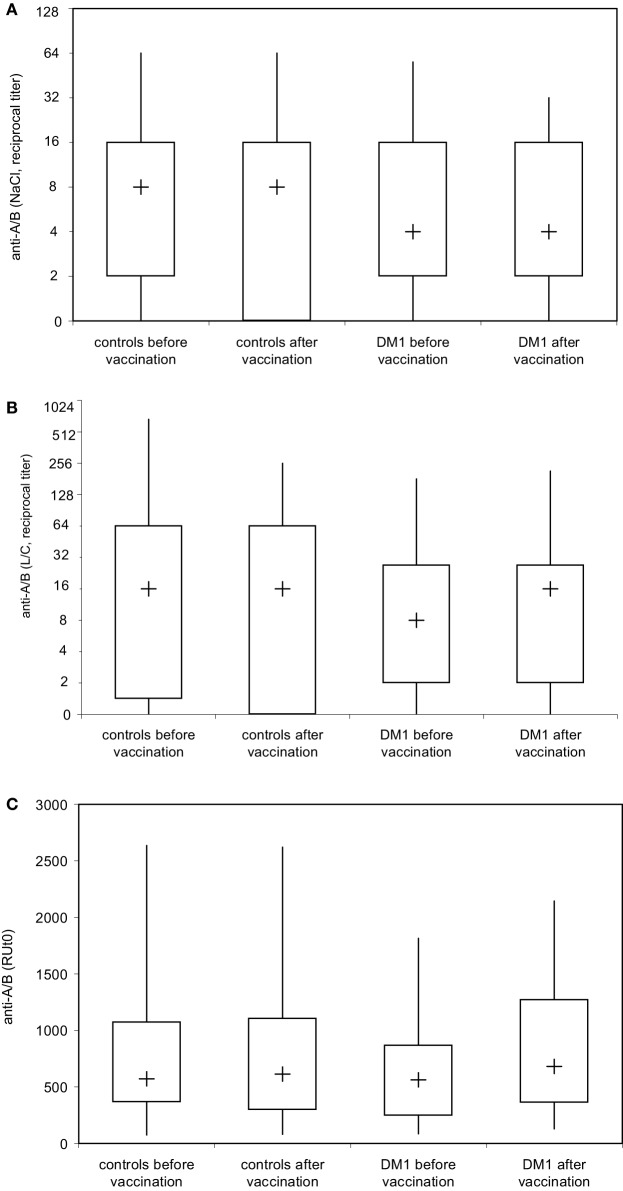
**Analysis of blood group anti-A and anti-B isoagglutinin binding to A and B trisaccharide antigen in real time by surface plasmon resonance (SPR) technology**. Serum samples of healthy individuals (*n* = 28) and patients with type I DM (*n* = 16) were serially twofold diluted with 0.9% saline solution and blood group anti-A and/or anti-B IgM **(A)** were determined by ID-Card 50520 (NaCl), while the ID-Card 50531 (LISS/Coombs) was used to determine IgG and IgM isoagglutinins **(B)**. The titer that led to visible agglutination of erythrocytes dispersed in the gel was selected positive. Alternatively **(C)**, serum samples diluted in HES-EP buffer (1:2) were injected over either the blood group A or blood group B trisaccharide-coupled CM5 sensor chip. Binding kinetics, i.e., association and dissociation were recorded as sensorgrams in resonance units (RU) against time in FC-I and FC-II, and the amount of anti-A/B antibody that associated with the blood group A/B trisaccharide antigen immobilized on the sensor chip surface was obtained by subtracting the FC-II value from the FC-I value and is given as resonance units (RU). Results are depicted using box plot diagrams, with the median represented by a cross, the interquartile range (IQR) represented by the box, and 5- and 95-percentile values represented by the whiskers; no statistically significant differences were found between study groups as calculated using the non-parametric two-tailed Mann–Whitney *U*-test.

To address whether immunization with Pn23 has an effect on blood group anti-A/B antibodies in patients with DM-type I as compared to healthy individuals, we first determined antibody titers in the DiaMed-ID Micro Typing System, using the ID-Card 50520 (NaCl) as well as the ID-Card 50531 (LISS/Coombs containing poly-specific AHG serum) to determine IgG in addition to IgM anti-A/B antibodies. We could observe no difference in blood group anti-A/B antibody titer prior and post immunization with Pn23 in both, healthy individuals and patients with DM-type I when the assay used was based upon erythrocyte aggregation in physiological NaCl (direct agglutination test, Figure 4A) or in the presence of anti-IgG Coombs serum (indirect agglutination test, Figure 4B). Interestingly, in a considerable percentage of study individuals of both groups (up to approximately 25%) the A/B isoagglutinin titers were lower than 1:4 in both agglutination systems, thus confirming previous findings of a relatively low sensitivity of the agglutination system to detect low isoagglutinin titers (6). To increase sensitivity, SPR technology was applied to further analyze anti-A/B antibodies, thus allowing real-time analysis of molecular interactions between antibodies and the isolated blood group trisaccharide antigen without molecular labeling leaving the structure of both molecules intact (6, 32). With this technology, biologically relevant antibody–antigen interactions can be assessed both quantitatively and qualitatively, as the carbohydrate antigens are presented in a physiological manner (11, 12, 31). Kinetics and affinity were investigated by analyzing the time curve and level of binding and concentration of anti-A/B antibodies in the sample by measuring mass binding, which further allowed the determination of the binding forces. The comparison of the response on the active and control surface allowed for the subtraction of bulk effects associated with buffer changes and for the high serum concentration with its mass binding, thus enabling the calculation of specific binding. Using SPR analysis, we found specific anti-A/B antibody binding to the respective blood group A/B trisaccharides in all individuals tested (Figure 4C), as has also been described previously for individuals with intact antibody responsiveness (6, 12). For all probes, a high association rate constant for the interaction and the use of a high ligand density on the sensor chip surface was found that promoted the mass transport limitation. As reference analyte with a known concentration, commercially available blood group anti-A or -B antibodies of IgM and blood group anti-A IgG were used (Figure 2, panel 1 shows monoclonal anti-A IgG as an example). For the example shown in Figure 2, panel 1, a sensor response of 1,000 RU corresponded to a shift of 0.1° in the SPR angle, which in turn correlates to approximately 16 μg/ml of anti-A IgG injected. Since the individuals selected for determination of *K*_D_ [one healthy control (Ind1) and one DM I patient (Ind2) as well as a control individual not vaccinated] contained sufficient amounts of anti-A IgG for affinity analysis with very low to undetectable anti-A IgM, titration was only performed for IgG anti-A/B antibody. Of note, SPR technology is capable to detect anti-A/B antibodies with values of 50–180 RU in serum samples where the microtube column assay showed no erythrocyte aggregation. Even with this sensitive detection system, no change in isoagglutinin titers was found following vaccination with 23-valent PnPs, neither in healthy individuals nor in DM1 patients (Figure 4C).

**Figure 5:**
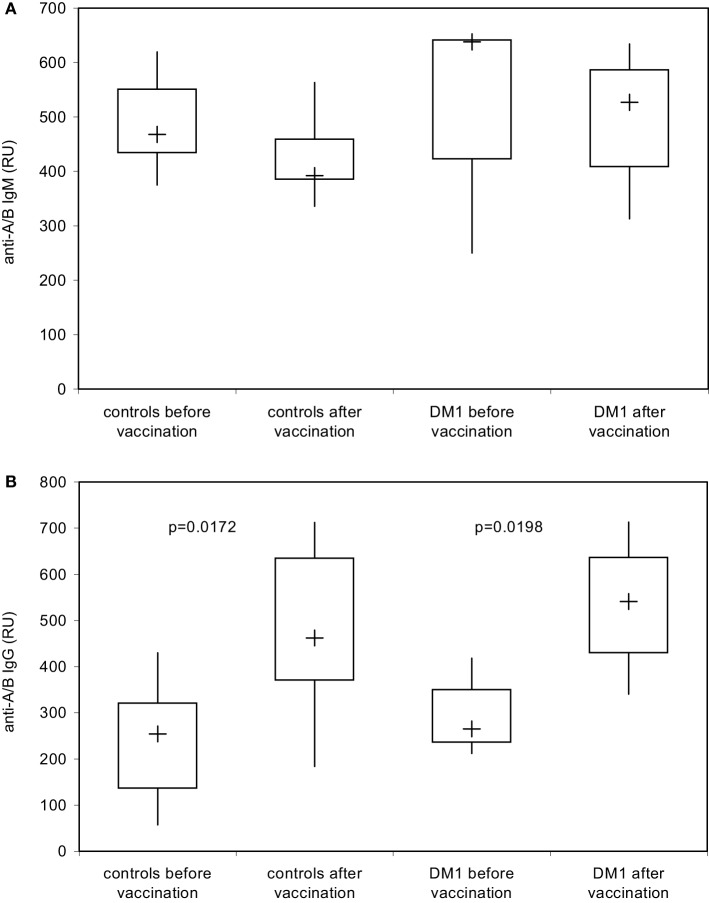
**Increase in blood group IgG anti-A/B antibodies in healthy individuals and in patients with type I DM after immunization with Pn23 vaccine as determined with the Biacore^®^ device**. To determine IgM **(A)** and IgG **(B)** anti-A/B antibodies in healthy individuals and patients with type I DM prior and post immunization with Pneumo 23 Vaccine “Pasteur Merieux” (Pn23), an anti-human IgM mAb **(A)** or anti-human IgG Abs **(B)** were applied as the second step reagents as described in Section “Materials and Methods.” Results are depicted using box plot diagrams, with the median represented by a cross, the interquartile range (IQR) represented by the box, and 5- and 95-percentile values represented by the whiskers; statistically significant differences between study groups were calculated using the non-parametric two-tailed Mann–Whitney *U*-test. For the IgM antibody response, no statistically significant differences were found between study groups.

Using the DiaMed-ID Micro Typing System in the direct and indirect agglutination assay as well as SPR technology under close to physiological conditions, both IgM- and IgG-antibodies were measured simultaneously, without discrimination between IgM and IgG response. To examine a possible booster effect of Pn23 vaccination on IgG anti-A/B antibodies only we applied isotype-specific second step antibodies to discriminate between blood group anti-A/B IgM and IgG antibodies. This system enabled us to detect an induction of blood group anti-A/B IgG (Figure 5B) but not IgM (Figure 5A) antibodies after immunization of healthy individuals with Pn23, an antibody response resembling a secondary/booster response. Interestingly, patients with type I DM showed a comparable induction of IgG isoagglutinins after immunization with Pn23 (Figure 5B), in agreement with their intact antibody responsiveness to pneumococcal polysaccharide, a T-independent antigen, while a significant impairment in primary antibody response to vaccination with T-cell-dependent antigens such as hepatitis A virus and diphtheria toxoid was described previously (17).

### Determination of Binding Characteristics of Anti-A/B Antibodies after Pneumovax^®^23 Immunization

**Figure 6:**
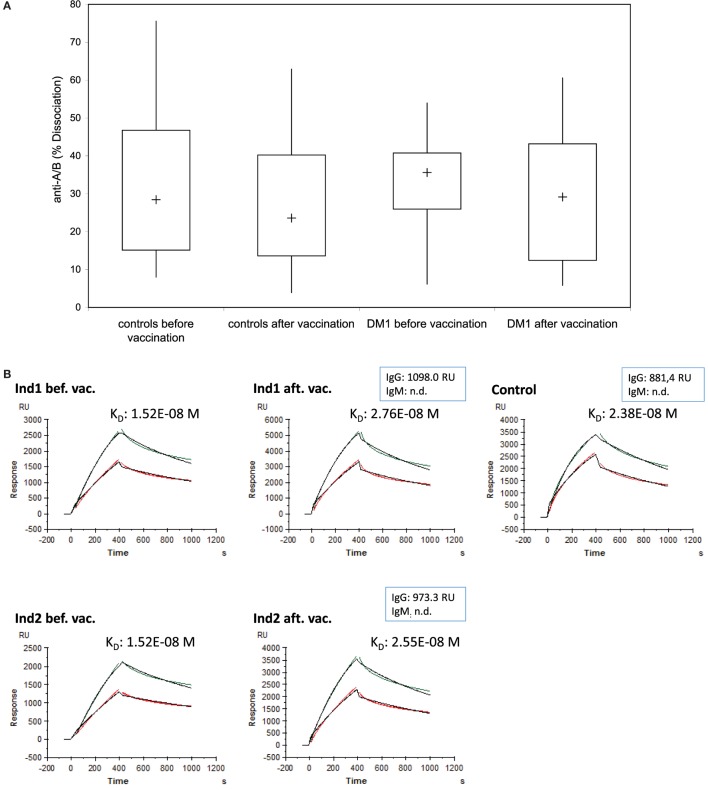
**Binding characteristics of anti-A/B antibodies after immunization with Pn23 vaccine**. The percentage of dissociation **(A)** of blood group anti-A/B antibodies before and after immunization with Pneumo 23 Vaccine “Pasteur Merieux” (Pn23) in healthy individuals (*n* = 28) and in patients with type I DM (*n* = 16) was investigated using the Biacore device, the CM5 chip, and the synthetic blood group specific A/B trisaccharides as described in Section “Materials and Methods.” Furthermore, the estimated affinity constant *K*_D_ **(B)** of blood group specific anti-A antibodies before and after pneumococcal vaccination were analyzed in serum samples of three healthy individuals and two patients with type I DM selected for their initially high anti-A antibody titer (>than 1:256 or >than 700 RU). Kinetic measurements of anti-A antibodies binding to surface bound trisaccharide before and after vaccination in two representative individuals (a healthy individual, Ind1, and a DM I patient, Ind2) who fulfilled these criteria are given; one healthy individual that was not included in the vaccination protocol but showed high titer for blood group anti-A antibodies was measured in parallel (Control). Green curves represent serum sample and red curves serum sample diluted by half; the black curves depict the calculated fit. n.d., not detectable.

To measure whether the induction of IgG-A/B antibodies observed after Pn23 immunization was accompanied by changes in binding characteristics of the blood group anti-A antibodies, as would be the case for a T-dependent IgG-booster response, we analyzed the binding dynamics of the anti-A/B antibodies as described above (Figure 2). We found no substantial change in the percentage of dissociation (Figure 6A) or the estimate of the affinity constant (*K*_D_, Figure 6B) of blood group-specific anti-A antibodies following pneumococcal vaccination, as calculated by BIAevaluation^®^ software from GE Healthcare. For estimation of the affinity constant *K*_D_, healthy individuals and patients with type I DM were selected that had initially high anti-A-antibody titers (>1:256 or >700 RU), associated with high anti-A IgG and low to undetectable anti-A IgM antibody levels. Only three healthy individuals and two patients with type I DM fulfilled this criterion. As shown in Figure 6 (depicting results from one healthy individual, Ind1 and one DM I patient, Ind2), our study gives evidence that immunization with Pn23 induces an increase in IgG anti-A/B antibody titers but has no effect on binding characteristics of blood group anti-A/B IgG antibodies, neither in the healthy individuals nor in the patients tested.

## Discussion

Based on previous findings (7, 13) it was our hypothesis that structural similarities in saccharide composition between pneumococcal polysaccharides and blood group A/B saccharide antigens could lead to production of cross-reactive blood group anti-A/B antibodies after immunization with capsular polysaccharide vaccines, because B cells could recognize shared carbohydrate epitopes (2, 8, 10, 33, 34). In the current study, we indeed show that immunization with Pn23 vaccine is followed by induction of blood group anti-A/B antibodies of the IgG isotype in healthy individuals. Anti-A/B IgG antibodies could only be demonstrated when SPR technology using the Biacore^®^ device was applied, while conventional technology employing erythrocyte aggregation assays using the DiaMed-ID Micro Typing System were unable to detect this side effect of Pn23 vaccination. Apparently, the increased sensitivity of SPR technology as compared to conventional erythrocyte agglutination assays as well as the near to physiological conditions of the molecular interaction between antibodies and the specific blood group trisaccharide antigen enabled us to detect the stimulation of small amounts of cross-reactive IgG antibodies (11, 12, 31, 32). A previous study described an alternative approach to measure isoagglutinins in an isotype-specific and sensitive manner by using synthetic blood group saccharides in ELISA and showed cross-reactivity between alpha-Gal and blood group B oligosaccharide (13). The increase in blood group anti-A/B IgG isotype response observed in our study provides additional evidence for a limited cross-reactivity between PnPs antigens and the carbohydrate moieties of blood group A/B trisaccharides. Whether the observed induction of IgG antibodies cross-reacting with A/B blood group antigens is of clinical relevance in healthy individuals remains to be determined but is unlikely due to the relatively small increase (approximately twofold) observed. Patients with type I DM displayed a comparable cross-reactive IgG antibody response against blood group antigens following Pn23 vaccination, although these patients present an autoimmune disease with profound immunological dysregulation (17, 18). Interestingly, cross-reactive IgM antibodies were not inducible, neither in DM1 patients nor in controls. This is characteristic of a booster response effecting IgG responses only and contrary to the effect of immunization with a conventional polysaccharide antigen such as PnPs inducing both anti-PnPs IgM and IgG antibody responses following booster vaccination (Figure 3).

An additional benefit of the SPR-based technology employed in this study is the capability for real-time measurements of affinity of antibody–antigen interactions without the use of molecular labeling, leaving the structure of both interacting molecules intact (11, 12, 32). Calculations of *K*_D_ as performed in our study using human serum as a sample can only be an estimate for the true affinity of a given antibody since we are dealing with a variety of polyclonal antibodies of different isotypes contributing to the overall affinity of the blood group antibodies detected in the SPR analysis used. Isotype bias has been limited since individuals with high blood group A IgG but low to undetectable blood group A IgM antibody were selected to estimate the binding affinity of anti-A IgG antibodies. To determine the affinity (*K*_D_) of serum antibodies of a single specificity, isolation and immobilization of the respective immunoglobulin clone would be required, which is only possible in individuals with monoclonal gammopathy of the desired specificity. Interestingly, we did not find a marked change in binding characteristics of the cross-reactive blood group anti-A/B antibodies, indicating that a relatively small increase in IgG antibody titer occurred without additional affinity maturation. An immune response to carbohydrate antigens such as to pneumococcal capsular polysaccharide serotypes 1, 2, 3, 4, 5, 6B, 7, 8, 9N, 9V, 10A, 11A, 12F, 14, 15B, 17F, 18C, 19A, 19F, 20, 22F, 23F, and 33F is thought to be generally TI, which can limit the B cell response to the production of IgM antibodies mainly and limited amounts of IgG antibodies, and the absence of affinity maturation of the rearranged antibody genes might lead to the expression of germline sequences of antibodies with limited affinity (5, 34, 35). In previous studies, the affinity of anti-carbohydrate antibodies for their antigens was shown to be 3–5 orders lower in magnitude than affinities of anti-protein or anti-peptide antibodies for their antigens (2, 3, 8, 35). In this study, however, we found no evidence for low affinity binding of blood group A/B antibodies obtained either from healthy individuals or from patients with type I DM. When a mixture of commercially available monoclonal blood group anti-A IgM and IgG antibodies was formulated, simulating the amount and composition of naturally occurring blood group anti-A antibodies, the dissociation rate was comparable to the serum samples analyzed in Figure 6 (data not shown). Germline antibodies recognizing carbohydrate epitopes might display significant cross-reactivity because the number of potential antigens the immune system must encounter appears to extensively outweigh the recombinational potential of the germline genes (2, 3, 34, 35). The recombinational potential of the germline genes for anti-Gal antibodies specific for blood group B substance in unimmunized individuals was shown to be limited, because anti-Gal antibodies are encoded by a group of 6–8 well-defined and structurally related germline progenitors in humans (36). Anti-Gal was shown to be the most abundant antibody in humans, constituting approximately 1% of immunoglobulins and found to be of IgG, IgM, and IgA isotype (37, 38). Cryptic antigens capable to bind anti-Gal are exposed on senescent human RBCs as well as on RBCs of patients with β-thalassemia and sickle cell anemia, but the amount of cryptic antigens expressed on RBCs was shown to be low (38). The preferential anti-Gal IgG subclass found by ELISA was IgG2 using Gal-specific synthetic saccharides, and these IgG2 antibodies showed cross-reactivity with blood group B antigen (13). ABO blood group antigens were shown to be the ideal targets to explore the concomitant effect of mutation on binding affinity and specificity of anti-carbohydrate antibodies (33). Mutant anti-carbohydrate antibodies with higher affinity achieved by single point mutations of specific residues lining the pocket on binding to the A and B blood group oligosaccharide antigens showed altered polarity, surface complementarity, and side chain aliphatic character, leading to improved binding affinity without loss of specificity (33). Furthermore, the poly-specificity in germline encoded antibodies to carbohydrate epitopes was shown to be due to greater conformational ability in their combining sites, and this flexibility helped to cope with new and changing pathogen surface structures by recognizing distinct highly conserved epitopes on bacterial polysaccharides (4, 33). Structural analysis of the affinity-matured antibody 48G7 and its germline precursor antibody showed that the germline precursor could undergo an induced fit upon binding to its cognate hapten, while the affinity-matured antibody 48G7 displayed lock and key binding. An immunological relationship between specific substances of ABO and type XIV pneumococcus gave further evidence for shared carbohydrate epitopes by showing that adsorption of the hemagglutinins in anti-type XIV horse serum with erythrocytes of each of the A/B blood groups removed the agglutinins not only of that group but also of the other groups (7). In addition to pneumococcus, other germs have been shown to cross-react with carbohydrate structures on the surface of human cells (39–43). The core oligosaccharides of low-molecular-weight LPS of pathogenic *Neisseria* spp. can mimic the carbohydrate moieties of glycosphingolipids (40). The core oligosaccharides of LPS of *Campylobacter jejuni* serotypes, which are associated with the development of Guillian–Barre’ syndrome, can exhibit mimicry of gangliosides (41). Finally, the O-chain of a number of *Helicobacter pylori* strains exhibit mimicry of Lewis(x) and Lewis(y) blood group antigens, giving evidence that molecular mimicry can serve to camouflage the bacterial surface from the host (42).

In conclusion, our study provides evidence for a limited carbohydrate epitope sharing between PnPs and blood group sugar epitopes in healthy individuals as well as in patients with type I DM that could be observed after Pn23 immunization. Cross-reactive anti-polysaccharide antibody responses were comparable between patients and controls with respect to the titer and the affinity. Blood group anti-A/B antibodies are considered to be naturally occurring antibodies that occur basically in any individual with an intact immune response also in the absence of antigenic encounter. The clinical relevance of the slight increase in blood group anti-A/B IgG isotype response in healthy individuals after Pn23 immunization is certainly limited; our findings however indicate modulation of anti-A/B IgG antibodies, e.g., a booster response during life not only by encounter with the genuine antigen such as incompatible blood transfusions or alloimmunization during pregnancy but also through molecular mimicry of particular carbohydrate epitopes shared by blood group AB substances and bacterial polysaccharides such as PnPs.

## Ethics Statement

The study was approved by the ethics committee of the Rudolfstiftung Hospital of the City of Vienna. Informed consent was obtained from every study participant.

## Author Contributions

MF and HW were the principal investigators, and they critically assessed data, performed statistical analysis, wrote the manuscript, and took primary responsibilities for the paper; WW and KS performed the experiments and analyzed the data; CB critically assessed data and was critically involved in the initial draft; NE-M conducted the clinical study and provided clinical data. All the authors critically participated in all revisions of the manuscript.

## Conflict of Interest Statement

The authors declare that the research was conducted in the absence of any commercial or financial relationships that could be construed as a potential conflict of interest.
